# Sequential Intravitreal Corticosteroid Rescue Including Ozurdex After Anti-VEGF Failure in Bilateral Uveitic Cystoid Macular Edema With Asymmetric Relapse

**DOI:** 10.7759/cureus.109008

**Published:** 2026-05-17

**Authors:** Ermal Simaku, Ahmed Nour Edien, Megi Popa

**Affiliations:** 1 Ophthalmology, University Hospital Mother Teresa, Tirana, ALB; 2 Ophthalmology, International Eye Clinic, Tirana, ALB

**Keywords:** anti-vegf failure, cystoid macular edema, dexamethasone intravitreal implant, ozurdex, triamcinolone acetonide, uveitic macular edema

## Abstract

Uveitic cystoid macular edema (CME) is a major cause of visual loss in patients with intraocular inflammation and may demonstrate inadequate response to anti-vascular endothelial growth factor (anti-VEGF) therapy. Intravitreal corticosteroids remain an important therapeutic option in inflammation-driven macular edema. This report describes the anatomical and functional course of bilateral uveitic CME refractory to anti-VEGF treatment and subsequently managed with sequential intravitreal corticosteroid therapy. A woman with bilateral uveitic CME was followed with serial optical coherence tomography and best-corrected visual acuity assessment. Initial evaluation showed marked bilateral macular thickening with reduced vision, more severe in the left eye. Despite repeated intravitreal anti-VEGF therapy, both anatomical and functional outcomes remained unsatisfactory, with persistent edema and visual deterioration. Treatment was then changed to bilateral intravitreal triamcinolone acetonide, after which substantial visual and anatomical improvement was observed in both eyes. The subsequent course was relapsing and asymmetric, with earlier recurrence in the left eye and later bilateral recurrence after missed follow-up. Because of the recurrent nature of the disease, bilateral dexamethasone intravitreal implant (Ozurdex) was administered, leading again to marked improvement in visual acuity and macular anatomy. This case demonstrates that bilateral uveitic CME refractory to anti-VEGF therapy may show limited response to repeated anti-VEGF treatment but substantial anatomical and functional improvement after sequential intravitreal corticosteroid therapy. It also highlights the relapsing and asymmetric course of inflammatory macular edema and the importance of individualized retreatment and close optical coherence tomography (OCT)-based monitoring.

## Introduction

Uveitic cystoid macular edema (CME) is one of the leading causes of visual impairment in patients with uveitis. The underlying pathophysiology is primarily inflammatory and involves breakdown of the blood-retinal barrier, resulting in accumulation of intraretinal fluid and cystoid spaces. Although anti-vascular endothelial growth factor (VEGF) therapy is effective in several retinal vascular disorders, its role in inflammation-driven macular edema is less predictable, particularly when inflammatory mediators rather than VEGF predominate [1-3]. Unlike diabetic or ischemic retinal disorders in which VEGF-mediated vascular leakage may be a dominant mechanism, uveitic CME is often driven primarily by inflammatory cytokines and immune-mediated blood-retinal barrier disruption.

Intravitreal corticosteroids can directly target ocular inflammation and reduce vascular permeability, making them an important treatment option in refractory uveitic CME [2,4,5]. Triamcinolone acetonide provides a relatively rapid intravitreal anti-inflammatory effect, whereas the dexamethasone intravitreal implant offers sustained steroid delivery and may be useful in recurrent or relapsing cases [2,4-7].

We report a case of bilateral uveitic CME with poor response to repeated anti-VEGF injections, followed by marked visual and anatomical improvement after stepwise intravitreal corticosteroid therapy using triamcinolone acetonide, followed by Ozurdex (dexamethasone intravitreal implant). This report illustrates the differential and relapsing bilateral behavior of uveitic CME despite symmetric treatment, as well as the anatomical and functional benefits of sequential intravitreal corticosteroid rescue after inadequate anti-VEGF response.

## Case presentation

**Figure 1:**
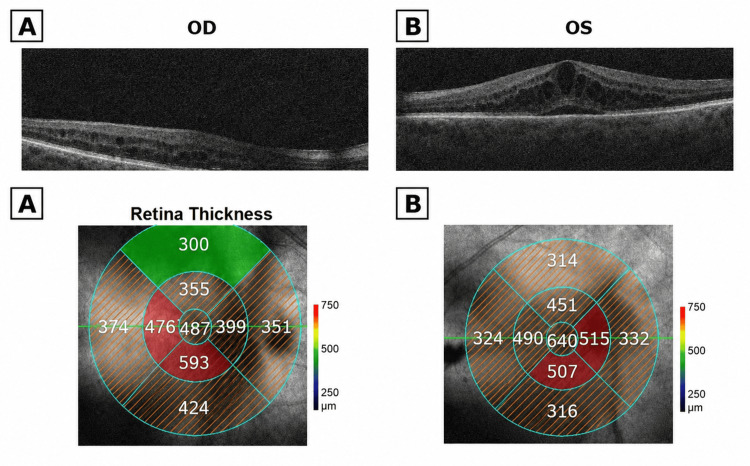
Baseline optical coherence tomography and retinal thickness maps. (A) Right eye (OD): macular OCT B-scan (top) and ETDRS retinal thickness map (bottom), demonstrating cystoid macular edema with increased central retinal thickness. (B): Left eye (OS): macular OCT B-scan (top) and ETDRS retinal thickness map (bottom), demonstrating cystoid macular edema with intraretinal fluid and increased retinal thickness. ETDRS: Early Treatment Diabetic Retinopathy Study

A 66-year-old woman, pseudophakic in both eyes, presented with bilateral decreased vision related to uveitic CME. At initial assessment on September 3, 2024, best-corrected visual acuity was 50 ETDRS (Early Treatment Diabetic Retinopathy Study) letters in the right eye (OD) and 20 ETDRS letters in the left eye (OS). These values were converted from decimal notation for uniform reporting. Spectral-domain optical coherence tomography (OCT) demonstrated bilateral CME with marked central thickening. Central foveal thickness (CFT) was 487 μm in OD and 640 μm in OS. IOP was 13 mmHg in her right eye and 12 mmHg in her left eye. The left eye showed more severe baseline involvement, which corresponded to poorer visual acuity (Figure 1).

Because of persistent bilateral CME, the patient underwent intravitreal anti-VEGF therapy. Three anti-VEGF injections were administered between October 2024 and March 2025. Despite treatment, the patient failed to achieve satisfactory functional or anatomical improvement, supporting the clinical impression that edema was predominantly inflammation-driven rather than primarily VEGF-driven.

**Figure 2:**
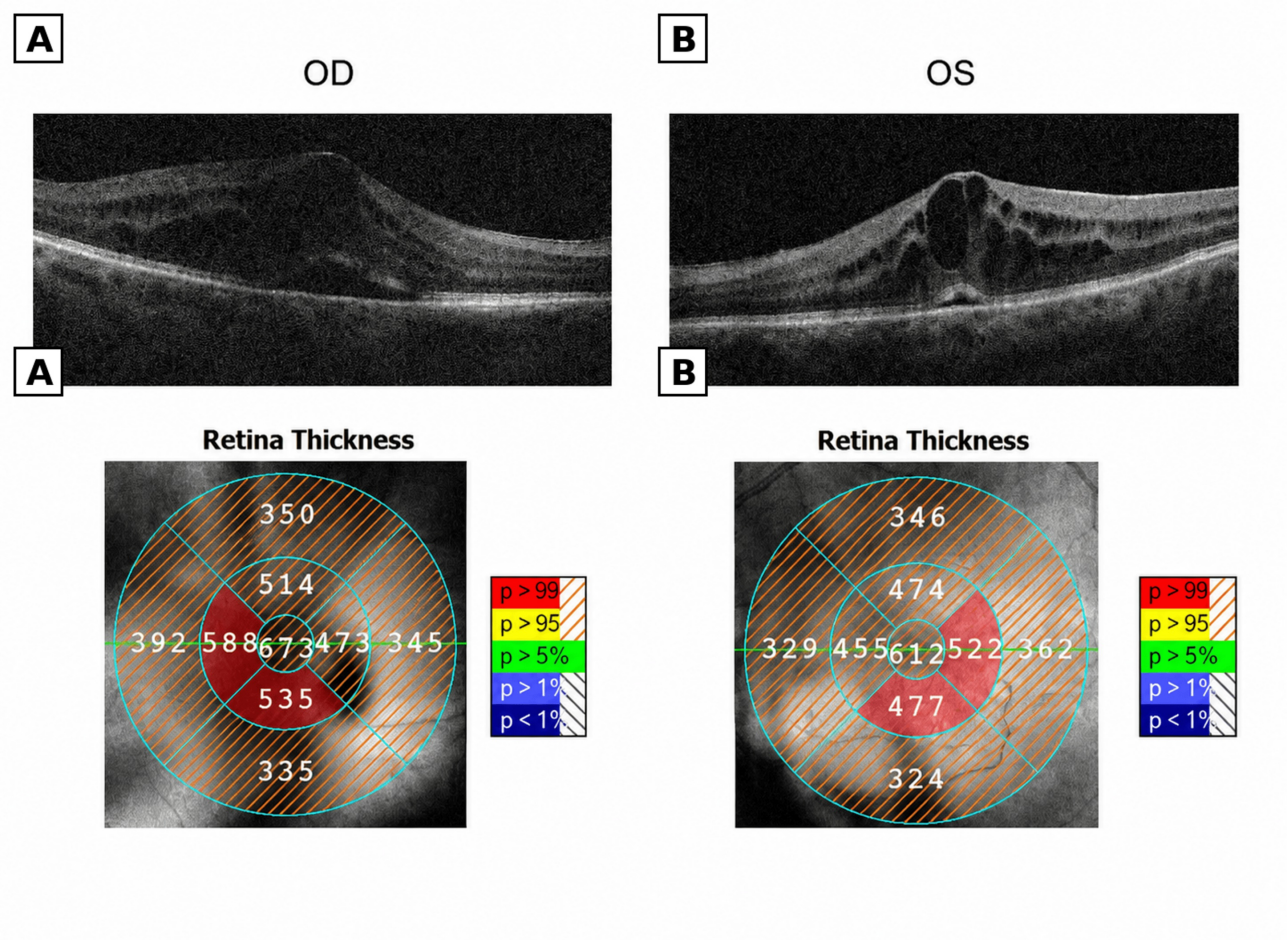
Optical coherence tomography and retinal thickness maps at month 1. (A) Right eye (OD): macular OCT B-scan (top) and ETDRS retinal thickness map (bottom), demonstrating persistent cystoid macular edema with increased central retinal thickness. (B) Left eye (OS): macular OCT B-scan (top) and ETDRS retinal thickness map (bottom), demonstrating prominent intraretinal cystoid spaces with persistent macular edema.

At reassessment on March 11, 2025, BCVA had deteriorated to 20 ETDRS letters in both eyes. OCT showed progression of edema, with CFT increasing to 673 μm in OD and 612 μm in OS. Intraretinal cystic changes remained prominent bilaterally (Figure 2).

**Figure 3:**
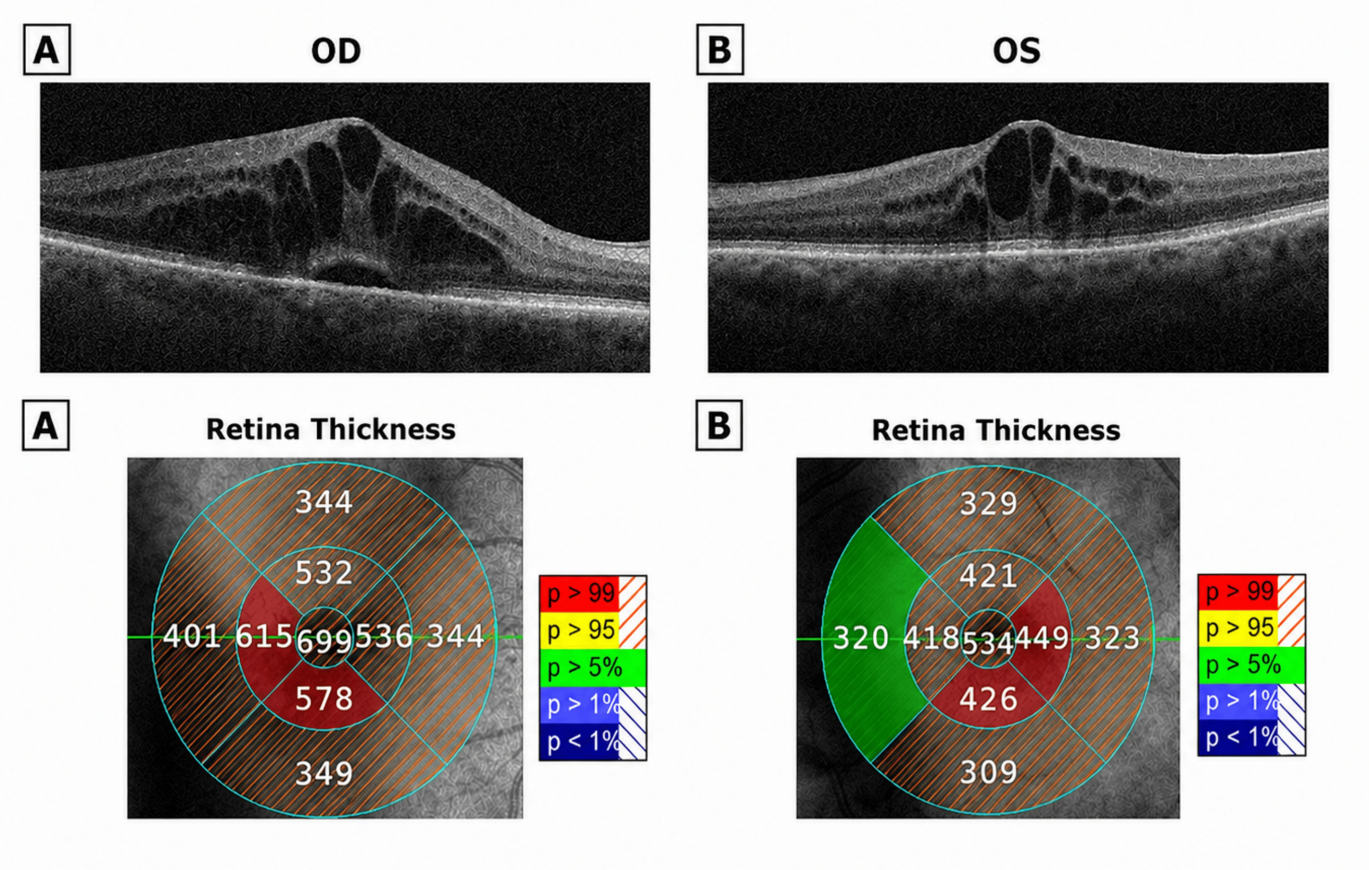
Optical coherence tomography and retinal thickness maps at month 3. (A) Right eye (OD): macular OCT B-scan (top) and ETDRS retinal thickness map (bottom), demonstrating worsening cystoid macular edema with increased central retinal thickness. (B) Left eye (OS): macular OCT B-scan (top) and ETDRS retinal thickness map (bottom), demonstrating persistent intraretinal cystoid spaces and progression of macular edema.

At follow-up on April 14, 2025, visual acuity remained poor, measuring 15 ETDRS letters in OD and 24 ETDRS letters in OS. OCT continued to show severe bilateral edema. Central foveal thickness reached 699 μm in OD and 535 μm in OS. Given the lack of meaningful response to anti-VEGF therapy, IOP with normal range in both eyes, and the presumed predominance of inflammatory pathophysiology, the treatment strategy was changed to bilateral intravitreal triamcinolone acetonide (Figure 3).

**Figure 4:**
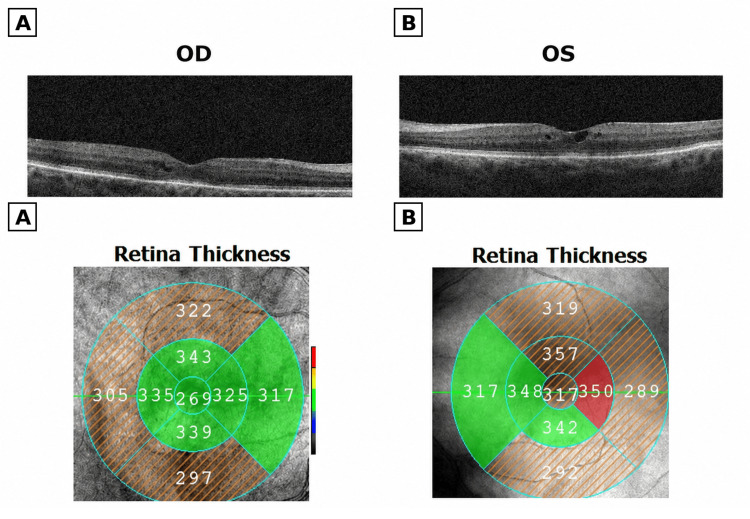
Optical coherence tomography and retinal thickness maps at month 6. (A) Right eye (OD): macular OCT B-scan (top) and EDTRS retinal thickness map (bottom), demonstrating near-complete resolution of cystoid macular edema with significant reduction in central retinal thickness. (B) Left eye (OS): macular OCT B-scan (top) and EDTRS retinal thickness map (bottom), demonstrating residual parafoveal thickening with marked anatomical improvement.

At the next visit on May 6, 2025, both the anatomical and functional responses were substantial. BCVA improved to 77 ETDRS letters in OD and 74 ETDRS letters in OS. Intraocular lens (IOL) remained within normal range, and no steroid-induced ocular hypertension was observed during follow-up. OCT demonstrated a marked reduction in intraretinal cystic spaces and normalization of foveal architecture compared with the previous examinations. CFT decreased to 269 μm in OD and 317 μm in OS (Figure 4).

**Figure 5:**
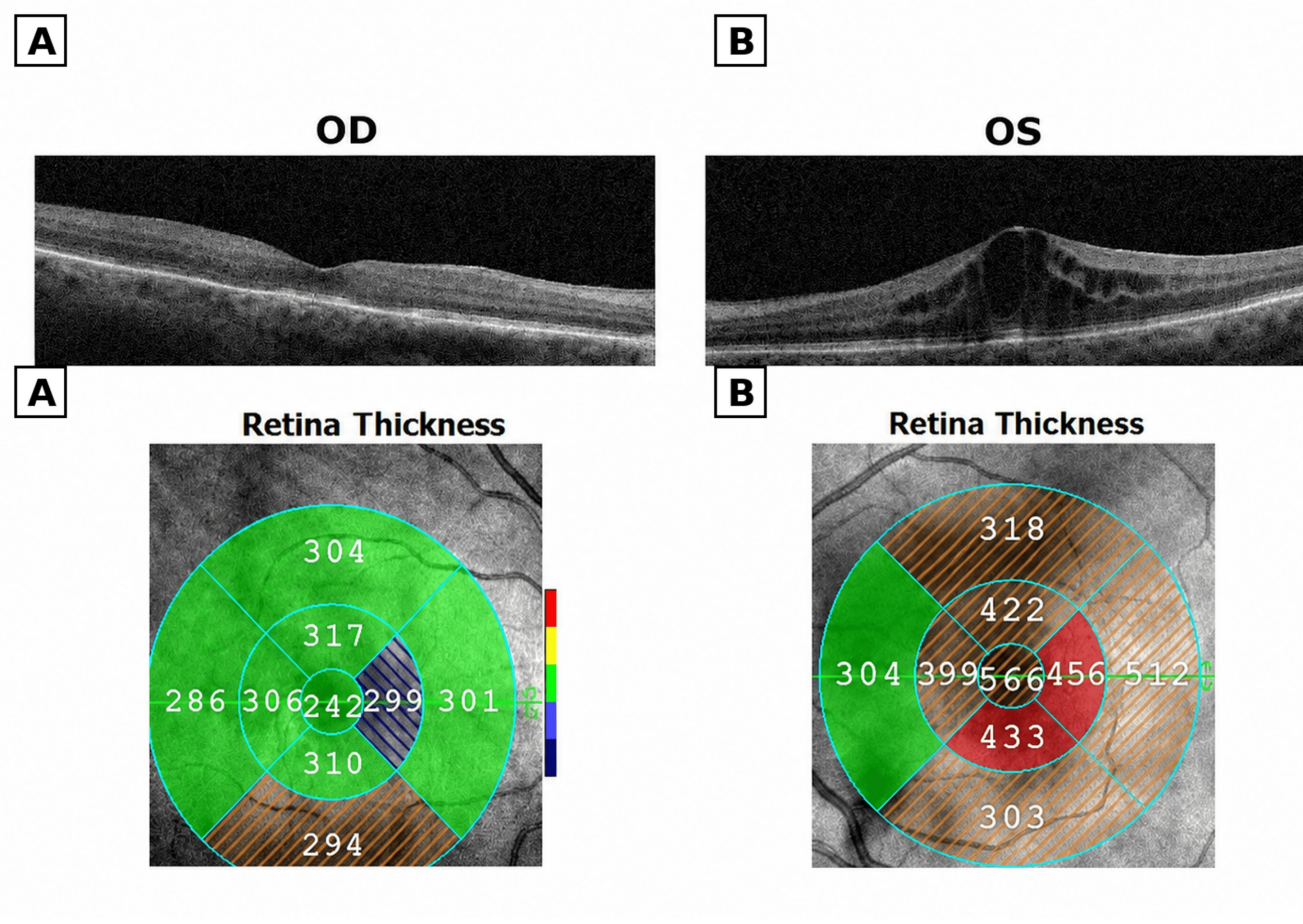
Optical coherence tomography and retinal thickness maps at month 9. (A) Right eye (OD): macular OCT B-scan (top) and ETDRS retinal thickness map (bottom), demonstrating maintained anatomical improvement with near-normal macular contour and retinal thickness. (B) Left eye (OS): macular OCT B-scan (top) and ETDRS retinal thickness map (bottom), demonstrating recurrent cystoid changes with persistent parafoveal retinal thickening.

On June 11, 2025, the right eye remained stable, with BCVA of 77 to 80 ETDRS letters, while the left eye showed recurrent visual deterioration to 20 ETDRS letters. OCT confirmed asymmetric recurrence, predominantly affecting OS, while OD remained relatively stable (Figure 5).

Because the disease course appeared relapsing and incompletely durable after the first steroid intervention, bilateral intravitreal triamcinolone acetonide was repeated. Following this second steroid treatment, the patient again improved clinically.

**Figure 6:**
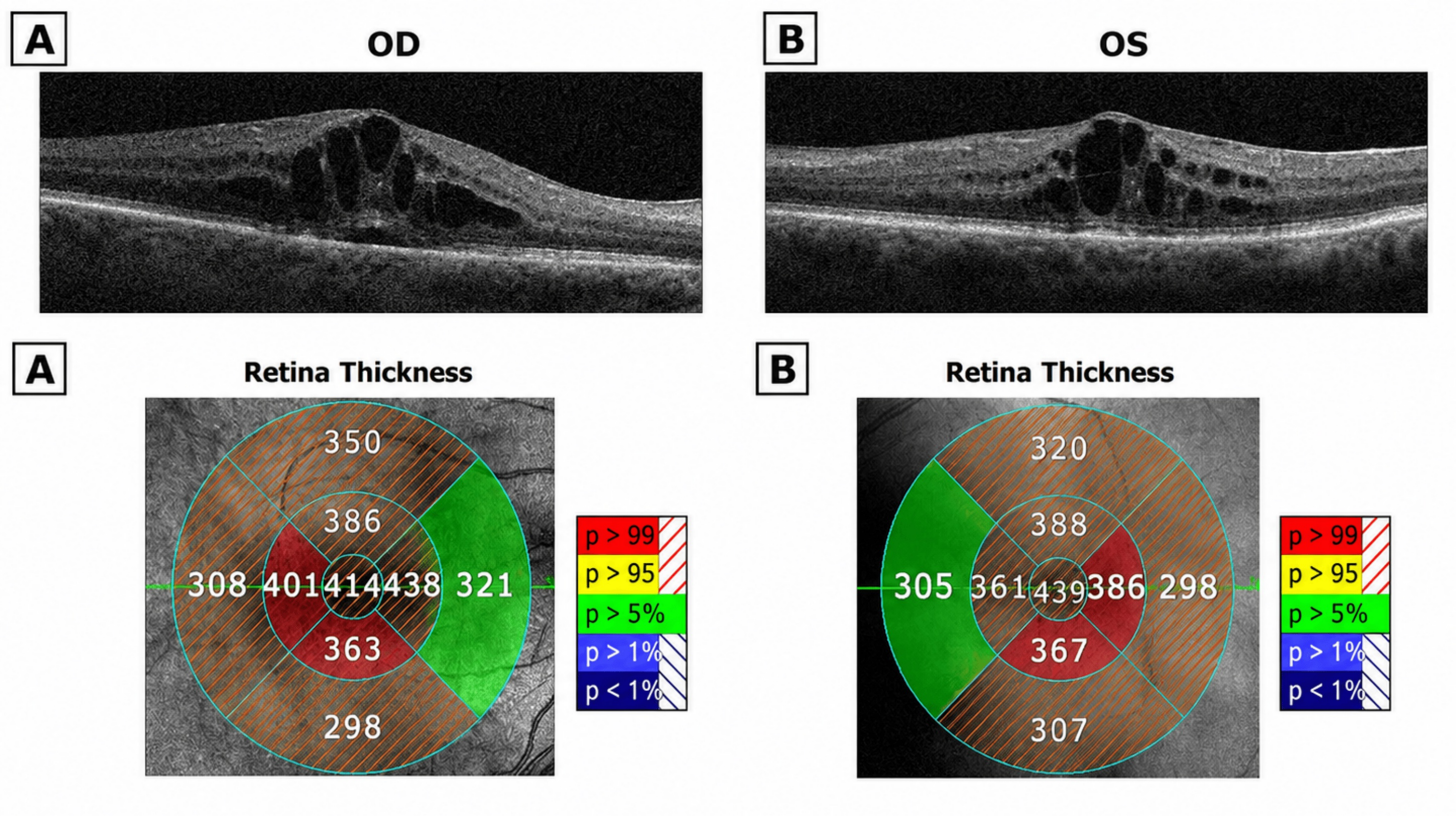
Optical coherence tomography and retinal thickness maps during asymmetric response. (A) Right eye (OD): macular OCT B-scan (top) and ETDRS retinal thickness map (bottom), demonstrating worsening retinal thickening with recurrence of macular edema changes. (B) Left eye (OS): macular OCT B-scan (top) and ETDRS retinal thickness map (bottom), demonstrating mild anatomical improvement with partial reduction in cystoid macular edema.

However, due to family-related circumstances, the patient did not return for the scheduled follow-up and re-presented only on November 26, 2025. At that time, BCVA had declined to 50 ETDRS letters in OD and 44 ETDRS letters in OS. IOP was 12 mmHg in her right eye and 17 mmHg in her left eye. OCT revealed recurrent bilateral CME. CFT measured 414 μm in OD and 439 μm in OS, confirming renewed anatomical worsening (Figure 6). 

**Figure 7:**
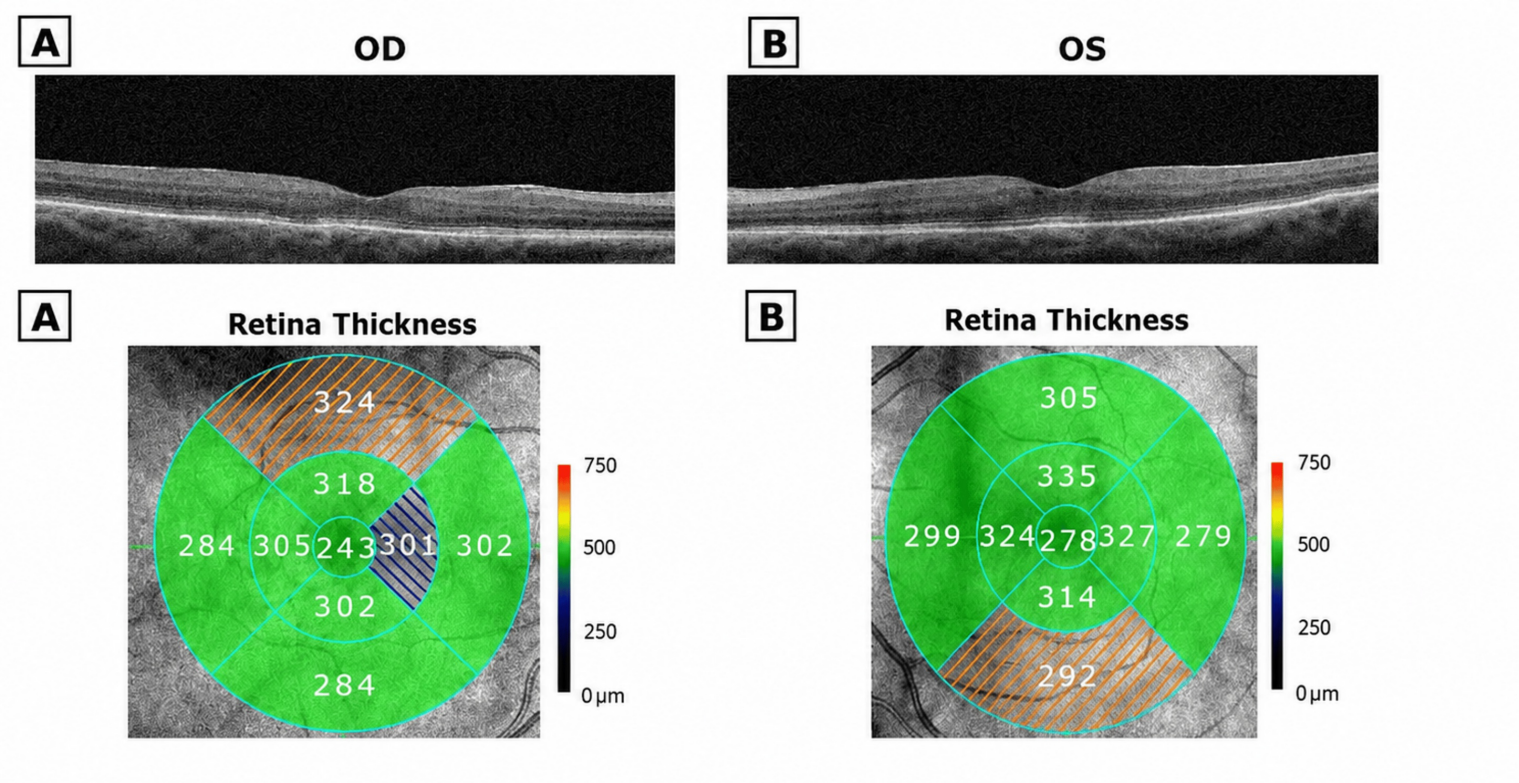
Optical coherence tomography and retinal thickness maps after dexamethasone intravitreal implant. (A) Right eye (OD): macular OCT B-scan (top) and ETDRS retinal thickness map (bottom), demonstrating complete resolution of cystoid macular edema with normalized retinal contour and thickness. (B): Left eye (OS): macular OCT B-scan (top) and ETDRS retinal thickness map (bottom), demonstrating a marked anatomical improvement with restoration of near-normal retinal architecture.

In view of recurrence after repeated triamcinolone and the need for a more sustained intravitreal corticosteroid effect, bilateral dexamethasone intravitreal implants were administered. At review on December 24, 2025, BCVA improved again to 77 ETDRS letters in OD and 76 ETDRS letters in OS. OCT demonstrated a marked reduction in edema, with restoration of a near-normal foveal contour. Final CFT was 243 μm in OD and 278 μm in OS (Figure 7).

IOP remained within normal range at each post-treatment visit. 

**Figure 8:**
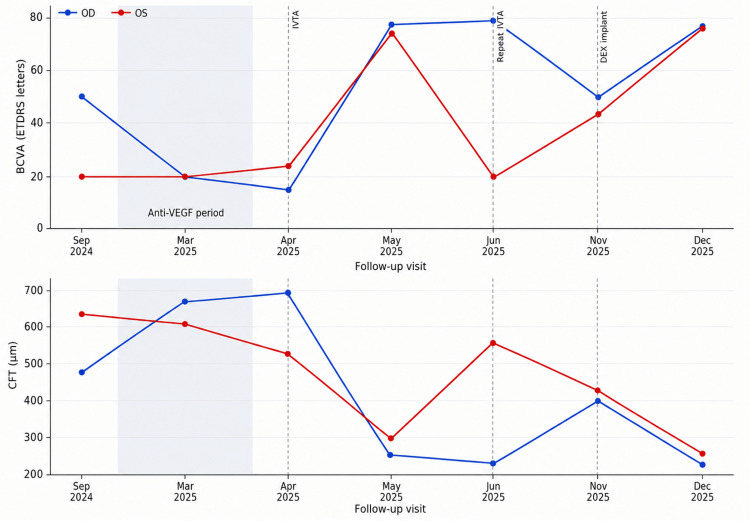
Functional and anatomical course over time following sequential intravitreal therapy. BCVA and central foveal thickness trends in both eyes across the treatment course, showing limited response after anti-VEGF therapy, marked improvement after intravitreal triamcinolone, recurrence, and subsequent improvement after dexamethasone implant. VEGF: vascular endothelial growth factor; BCVA: best-corrected visual acuity; OD: right eye; OS: left eye; ETDRS: Early Treatment Diabetic Retinopathy Study; IVTA: intravitreal triamcinolone; DEX implant: dexamethasone implant; CFT: central foveal thickness

Overall, the serial OCT findings documented a fluctuating but treatment-responsive course. The major anatomical and functional trends are illustrated in Figure 8 and summarized in Table 1. 

**Table 1:** Longitudinal anatomical and functional course of bilateral uveitic cystoid macular edema. CME: cystoid macular edema; VEGF: vascular endothelial growth factor; BCVA: best-corrected visual acuity; OD; right eye; OS: left eye; CFT: central foveal thickness; ETDRS: Early Treatment Diabetic Retinopathy Study

Date	Treatment/clinical stage	BCVA OD	BCVA OS	CFT OD	CFT OS	Clinical interpretation
September 3, 2024	Baseline assessment	50 ETDRS	20 ETDRS	487 μm	640 μm	Bilateral uveitic CME, more severe in OS
March 11, 2025	After three anti-VEGF injections	20 ETDRS	20 ETDRS	673 μm	612 μm	Persistent/worsening CME despite anti-VEGF
April 14, 2025	Before the corticosteroid switch	15 ETDRS	24 ETDRS	699 μm	535 μm	Severe persistent bilateral edema
May 6, 2025	After bilateral intravitreal triamcinolone	77 ETDRS	74 ETDRS	269 μm	317 μm	Marked anatomical and functional improvement
June 11, 2025	Asymmetric relapse	77-80 ETDRS	20 ETDRS	242 μm	566 μm	OD stable; early severe relapse in OS
November 26, 2025	After a missed follow-up	50 ETDRS	44 ETDRS	414 μm	439 μm	Recurrent bilateral CME
December 24, 2025	After bilateral Ozurdex	77 ETDRS	76 ETDRS	243 μm	278 μm	Marked improvement after sustained steroid therapy

## Discussion

This case illustrates several clinically relevant aspects of bilateral uveitic CME, including anti-VEGF insufficiency, corticosteroid responsiveness, relapse after steroid effect waning, asymmetric inter-eye behavior, and the need for OCT-guided retreatment.

First, it highlights the limitations of anti-VEGF therapy in inflammation-driven macular edema. Although anti-VEGF agents can reduce vascular permeability, their effect may be incomplete when inflammatory mediators are the dominant pathogenic mechanism. In the present case, three anti-VEGF injections administered over several months failed to produce meaningful visual recovery and did not prevent anatomical worsening. Both BCVA and OCT parameters deteriorated during this period, suggesting insufficient control of the underlying inflammatory process [3].

Second, the case shows a pronounced response to intravitreal corticosteroids. Following bilateral triamcinolone acetonide injection, the patient experienced substantial improvement in both anatomical and functional outcomes. This rapid response supports the view that inflammation was the principal driver of edema [2,4,5]. The reduction in CFT from 699 μm to 269 μm in the right eye and from 535 μm to 317 μm in the left eye was accompanied by major visual gain, reinforcing the therapeutic relevance of steroid-based treatment in selected patients with uveitic CME. Expressed as an anatomical change, CFT decreased by approximately 61% in OD and 41% in OS after triamcinolone, paralleling the improvement in BCVA.

Similar findings have been reported in previous studies evaluating intravitreal corticosteroid therapy for uveitic macular edema. Dexamethasone intravitreal implant has been shown to provide meaningful anatomical and functional improvement in noninfectious uveitic macular edema, including cases with recurrent or chronic disease requiring sustained intraocular anti-inflammatory control [2,4-7]. In the available literature, reduction in central retinal thickness and improvement in visual acuity after Ozurdex support the concept that corticosteroid-based therapy directly addresses the inflammatory component of uveitic CME more effectively than VEGF suppression alone in selected patients [2,4,5]. Compared with these reports, the present case is notable for its bilateral involvement, poor response to repeated anti-VEGF injections, rapid response after intravitreal triamcinolone, asymmetric relapse between the two eyes, and subsequent bilateral improvement after dexamethasone implant. This sequence reinforces the importance of individualized treatment escalation and eye-specific OCT-guided retreatment in relapsing inflammatory macular edema.

Third, the disease course was relapsing and asymmetric. Although both eyes initially improved after triamcinolone, the left eye relapsed earlier and more severely. This asymmetry is clinically important because bilateral inflammatory retinal disease does not always behave symmetrically, even when the same therapeutic approach is used in both eyes. Such cases require individualized retreatment decisions based on separate eye-specific clinical and OCT findings rather than assuming parallel evolution. The relapse observed during follow-up may be explained by the chronic and recurrent nature of uveitic macular edema, the limited duration of action of intravitreal triamcinolone, and the persistent underlying inflammatory drive. In addition, the missed follow-up interval likely delayed detection and retreatment of recurrent edema, allowing anatomical worsening before the patient re-presented. The asymmetric recurrence, with earlier and more severe relapse in the left eye, may reflect inter-eye differences in inflammatory activity, baseline disease severity, or steroid responsiveness.

Fourth, the case demonstrates the practical value of therapeutic sequencing. Repeated triamcinolone achieved renewed improvement, but recurrent disease and the need for a longer-lasting intravitreal anti-inflammatory effect justified escalation to Ozurdex (dexamethasone intravitreal implant). The favorable response after dexamethasone implantation supports the use of sustained corticosteroid delivery in recurrent or insufficiently durable cases [2,4-7].

Another important lesson from this case is the role of close OCT-based follow-up. The serial OCT examinations documented not only baseline severity and treatment response but also recurrence patterns over time. In real-world practice, missed visits can delay recognition of relapse and allow recurrent edema to progress. In this patient, the prolonged absence from follow-up was associated with significant visual and anatomical worsening by November 2025. This underscores the need for regular monitoring, especially in patients with previously recurrent CME. Because intravitreal corticosteroids can be associated with adverse effects, including intraocular pressure elevation and cataract progression in phakic eyes, lens status and IOP monitoring should also be incorporated into long-term management decisions.

The differential diagnosis included other causes of bilateral CME, including diabetic macular edema, retinal vein occlusion-related macular edema, pseudophakic Irvine-Gass syndrome, and medication-related or inflammatory macular edema. However, the bilateral presentation, clinical context of uveitis, poor response to repeated anti-VEGF injections, and marked response to intravitreal corticosteroid therapy supported an inflammation-driven mechanism. No clinical features suggesting retinal vein occlusion or diabetic macular edema as the primary cause were identified.

The main limitation of this report is that it describes a single patient, and the findings cannot be generalized without caution. In addition, as in many real-world cases, follow-up intervals were influenced by patient circumstances rather than a rigid treatment protocol. Nevertheless, the case remains valuable because it documents a clear pattern of anti-VEGF insufficiency, corticosteroid responsiveness, recurrence, and renewed response to sustained steroid therapy.

## Conclusions

Bilateral uveitic CME may respond poorly to repeated anti-VEGF injections when the inflammatory component predominates. In this patient, sequential intravitreal corticosteroid therapy with triamcinolone acetonide followed by Ozurdex (dexamethasone intravitreal implant) resulted in major anatomical and functional improvement after anti-VEGF failure. The case also emphasizes the relapsing and asymmetric nature of uveitic CME and the importance of individualized retreatment strategies guided by serial OCT monitoring. Continued monitoring for recurrence and corticosteroid-related adverse effects, particularly IOP elevation, remains important during long-term management.
